# Valency and Binding Affinity Variations Can Regulate the Multilayered Organization of Protein Condensates with Many Components

**DOI:** 10.3390/biom11020278

**Published:** 2021-02-14

**Authors:** Ignacio Sanchez-Burgos, Jorge R. Espinosa, Jerelle A. Joseph, Rosana Collepardo-Guevara

**Affiliations:** 1 Maxwell Centre, Cavendish Laboratory, Department of Physics, University of Cambridge, J J Thomson Avenue, Cambridge CB3 0HE, UK; is490@cam.ac.uk (I.S.-B.); jr752@cam.ac.uk (J.R.E.); jaj52@cam.ac.uk (J.A.J.); 2 Yusuf Hamied Department of Chemistry, University of Cambridge, Lensfield Road, Cambridge CB2 1EW, UK; 3 Department of Genetics, University of Cambridge, Downing Site, Cambridge CB2 3EH, UK

**Keywords:** protein liquid–liquid phase separation, multicomponent condensates, minimal protein model, multilayered condensates, multiphase condensates

## Abstract

Biomolecular condensates, which assemble via the process of liquid–liquid phase separation (LLPS), are multicomponent compartments found ubiquitously inside cells. Experiments and simulations have shown that biomolecular condensates with many components can exhibit multilayered organizations. Using a minimal coarse-grained model for interacting multivalent proteins, we investigate the thermodynamic parameters governing the formation of multilayered condensates through changes in protein valency and binding affinity. We focus on multicomponent condensates formed by scaffold proteins (high-valency proteins that can phase separate on their own via homotypic interactions) and clients (proteins recruited to condensates via heterotypic scaffold–client interactions). We demonstrate that higher valency species are sequestered to the center of the multicomponent condensates, while lower valency proteins cluster towards the condensate interface. Such multilayered condensate architecture maximizes the density of LLPS-stabilizing molecular interactions, while simultaneously reducing the surface tension of the condensates. In addition, multilayered condensates exhibit rapid exchanges of low valency proteins in and out, while keeping higher valency proteins—the key biomolecules involved in condensate nucleation—mostly within. We also demonstrate how modulating the binding affinities among the different proteins in a multicomponent condensate can significantly transform its multilayered structure, and even trigger fission of a condensate into multiple droplets with different compositions.

## 1. Introduction

Liquid–liquid phase separation (LLPS) is one of the key processes employed by cells to control the spatiotemporal organization of their many components, i.e., via formation and dissolution of biomolecular condensates. These condensates are liquid-like membraneless compartments highly enriched in specific biomolecules (e.g., proteins and RNAs) and depleted in others [1,2,3,4]. Because of their ability to selectively concentrate and exclude biomolecules, these condensates have been suggested to act as reaction crucibles that speedup chemical reactions, or sequestrate unwanted components to prevent/promote reactions within the cytoplasm and nucleoplasm [4]. In addition, biomolecular condensates play important roles in biological processes, such as, signaling [5,6], chromatin reorganization[7], formation of super-enhancers [8], buffering cellular noise [9], and many others [10,11,12,13,14,15,16,17,18,19]. Moreover, misregulated LLPS inside cells is associated with the development of degenerative diseases, aging-related pathologies (e.g., Alzheimer’s disease [20,21], Parkinson’s disease [22,23], amyotrophic lateral sclerosis [24]) and cancers[14]. Thus, elucidating the factors that tune the stability, structure, and ultimately function of condensates is highly desirable.

Formation of biomolecular condensates via LLPS is a collective phenomenon emerging from the dynamic formation and breakage of thousands of weak attractive interactions among multivalent proteins—often involving both intrinsically disordered regions (IDRs) [25,26,27,28] and globular domains [29,30] and, in many cases, RNAs [31,32,33,34,35,36,37,38,39]. Biomolecules that are essential to the formation of condensates are termed “scaffolds”, while those that are recruited to condensates through their interactions with the scaffolds are known as “clients” [40]. Above a critical scaffold concentration and under conditions that favor intermolecular interactions (e.g., in temperature, pH, salt), the enthalpy gain stemming from high density multivalent biomolecular interactions among scaffolds becomes sufficient to compensate for the entropy loss due to demixing, and the system undergoes a phase transition[41]; thereby, demixing into a protein-enriched condensed liquid phase (i.e., biomolecular condensate) and a surrounding protein-depleted liquid phase occurs [2,42,43,44].

Despite being condensed liquids, biomolecular condensates are not exclusively homogeneous systems. Indeed, multilayered or multiphase organizations (multiple coexisting liquid or solid phases within individual condensates) have been observed in stress granules [39,45], the nucleoli [15], and nuclear speckles [46]. In vitro, complex coacervates [33,47,48] and mixtures of RNA-binding proteins and RNA molecules [34,49] also form hierarchically organized condensates with various coexisting phases or layers. Experiments and simulations propose that the multilayered organization of the nucleolus emerges from differences in the surface tensions of the various phases [15]. The importance of surface tension in driving a multilayered molecular organization of condensates has been corroborated for in vitro complex coacervates that form condensates with up to three layers [47,50]. Competing interactions among protein–RNA networks also play a role in driving the formation of multiphase condensates [33]. Simulations and mean-field theory further explain that multicomponent systems separate into multiple coexisting liquid phases when their components bind to one another with sufficiently different binding affinities [51,52].

To further understand some of the thermodynamic origins that drive multicomponent condensates to adopt multilayered structures, here we investigate how the variance in the valencies and binding affinities among phase-separating proteins impact the internal structure, composition, and interfacial free energy of multicomponent biomolecular condensates. Molecular simulations provide useful tools for investigating this question: they enable us to control key properties of phase-separating proteins (e.g., valency and binding affinity), and subsequently gain a detailed molecular picture of the internal organization of the ensuing multicomponent condensates. Using a minimal coarse-grained model for multivalent proteins [41,53] further allows us to simulate the formation of biomolecular condensates with many components while simultaneously measuring thermodynamic parameters that we hypothesize could explain the emergent condensate organization. We conduct molecular dynamics simulations of multicomponent protein mixtures using our minimal protein model [41,53] and find that the variations in valency and binding affinities among protein components determine whether the system forms a multilayered condensate (with spatially segregated components within) or multiple non-interacting condensates with different compositions. Our simulations propose that a major physical determinant explaining the role of valency and binding affinity in the emergence of multilayered condensate organization is the combined minimization of the interfacial free energy and maximization of the condensate liquid-network connectivity. Furthermore, our work suggests that chemical modifications, which can modulate the relative valency or binding affinities among a small subset of key proteins within a multicomponent condensate, can be used to favor the emergence of one or various multilayered condensates on demand [54,55].

## 2. Methods

The highest attainable resolution to investigate the process of protein condensation with computer simulations is now approaching that of atomistic models [56,57,58]. However, since LLPS is a collective phenomenon that involves thousands of interacting biomolecules [41,42], coarse-grained models with different levels of resolution are still the most effective potentials to decipher the molecular and biophysical forces driving protein demixing and self-assembly [34,53,58,59,60,61,62,63,64,65,66,67,68,69,70,71,72,73,74]. Indeed, coarse-grained simulation studies have been useful at linking a wide-range of protein characteristics to the modulation of their phase behavior—e.g., valency [39,65,75,76,77,78], topological distribution of binding sites [53], amino acid sequence and patterning [79,80,81,82], IDR conformation [69,83]—and the emergence of multilayered condensate organizations [15,33].

**Figure 1:**
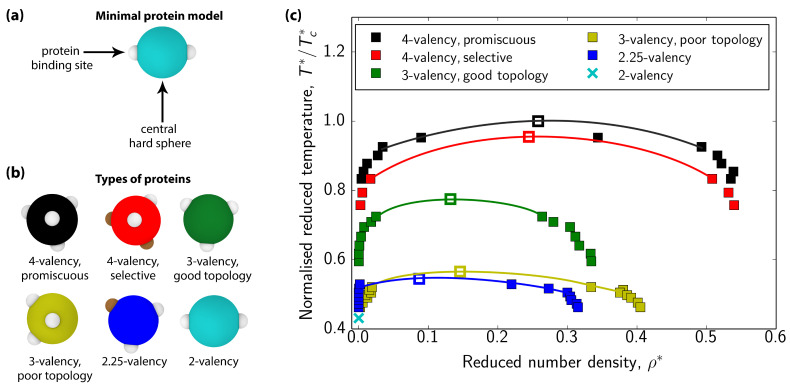
(**a**) Minimal protein model. In our coarse-grained model, proteins are represented by hard-spheres with different numbers/types of binding sites on their surface. (**b**) Schematic representation of the different multivalent proteins studied in this work. 4-valency promiscuous proteins have four promiscuous binding sites in a tetrahedral arrangement. 4-valency selective proteins have two types of binding sites that interact exclusively with those of the same type on their parent protein (i.e., selective homotypic binding), and promiscuously with any site on their non-parent proteins. In total, this protein has four patches arranged in a tetrahedral topology. 3-valency good topology proteins have three promiscuous binding sites separated by angles of $120^{\circ }$ in a plane, which minimizes the steric hindrance for binding. 3-valency ‘poor’ topology proteins have binding sites separated by $90^{\circ }$ angles, which leads to larger steric hindrance. 2.25-valency proteins possess the same topology as the 3-valency good topology proteins, but the strength of one of the binding sites (brown) is decreased to 1/4 of the net strength interaction of the other two sites (i.e., brown–brown = 25% white–white). Interactions between that special site and regular sites follow Lorentz-Berthelot combination rule ($\frac{\epsilon_{1}+\epsilon_{2}}{2}$). 2-valency proteins have two binding sites in a polar arrangement. These snapshots and subsequent ones were rendered using OVITO [85]. (**c**) Phase diagrams in the ($T^{*}/T_{c}^{*}$) $-\rho^{*}$ plane for the 6 different proteins considered in this study. $T^{*}/T_{c}^{*}$ is the reduced temperature normalized by the highest critical reduced temperature [corresponding to the 4-valency promiscuous protein ($T_{c}^{*}$ = 0.121)] and $\rho^{*}$ is the reduced density. Filled squares represent the coexistence points computed using Direct Coexistence simulations. Empty squares depict the estimated critical points using the universal scaling of the coexistence densities near the critical point, and the law of rectilinear diameters [86]. Further details on the reduced units used in this work are given in the Appendix A. Note that the 2-valency protein does not phase separate on its own (cyan cross).

We describe multivalent proteins using a patchy particle model, following Refs. [31,41,53]. Specifically, a single protein is represented by a hard-sphere decorated with sticky patches that act as binding sites [Figure 1a]; hence, the number of patches is related to the effective valency of the protein. By modifying the valency, the topological distribution of patches on the protein surface, and the specificity of the interactions among patches, we can investigate the phase behavior of different types of multivalent proteins. A great advantage of this model is that, due to its simplicity, it can be used to simulate systems containing thousands of interacting proteins, and concurrently, compute phase diagrams of multicomponent multivalent protein systems through the direct coexistence method [31,41,53]. Further details on our simulations are given in the Appendix A. Despite the model limitations, such as its inability to describe amino acid sequence effects, multivalent binding between two proteins or other fine molecular details of proteins, we have previously shown [41] that this simple approach captures well the dependency of the critical solution parameters on protein valency, observed in experiments [76,84] and recapitulated by a sequence-dependent model [80]. Figure 1 depicts the different types of proteins that comprise our multicomponent mixtures and their corresponding phase diagrams as single-component systems.

Our previous work [41,53] demonstrated that proteins with higher valencies that can phase separate via homotypic interactions are characterized by higher critical points in their phase diagrams as pure systems [Figure 1c], and have higher concentrations within the multicomponent condensates [41]. Hence, when all these proteins are mixed together, the highest valency species (i.e., the two types of 4-valency proteins) are expected to behave as the scaffolds (i.e., the set of biomolecules that drive LLPS), while the lower valency species [see Figure 1b] act as clients that are incorporated into condensates via their interactions with the scaffold proteins.

## 3. Results

### 3.1. Impact of Protein Valency and Binding Affinity in the Molecular Organization of Multicomponent Condensates

Intracellular biomolecular condensates are typically highly multicomponent systems formed by tens to several thousands of different biomolecules (e.g., proteins and nucleic acids), potentially spanning a plethora of valencies and binding affinities [2,40,81,87]. Therefore, the first question we ask is: how do variations in the valency and binding affinity among proteins in multicomponent mixtures impact the internal molecular organization of the condensates they form? To this end, we use our minimal protein model to perform direct coexistence simulations [40,88,89] of different multicomponent protein mixtures, in the NVT ensemble at a fixed temperature below the critical one.

**Figure 2:**
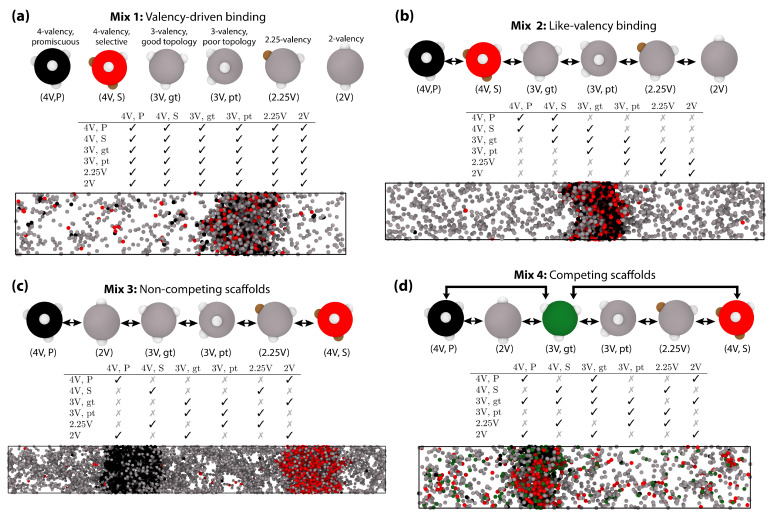
(**a**) Valency-driven binding. In this system, all interactions between different protein types are enabled; the table inset describes which proteins bind to one another with high-affinity (tick) or low-affinity (cross). A representative snapshot of the coexisting condensate with the dilute phase at $T^{*}=0.09$ taken from a Direct Coexistence (DC) simulation is shown. Here, 4-valency promiscuous binding proteins and 4-valency selective binding ones are colored in black and red respectively, while lower valency proteins are colored in grey (as shown in the snapshots, top panel). (**b**) Like-valency binding. In this system, proteins participate in either homotypic binding or interact with other proteins of like-valency, as indicated in the table inset. A snapshot of this system at $T^{*}=0.09$ is shown, with the same color code as in (**a**). (**c**) Non-competing scaffolds. Homotypic interactions are allowed (see diagonal of the table inset) plus the heterotypic interactions indicated in the table. In this case, the two highest valency proteins (i.e., 4-valency promiscuous and 4-valency selective) cannot bind to each other. For this interaction scheme, two different condensates with different compositions are formed in our DC simulations at $T^{*}=0.083$. Note that for this system, no phase separation was observed at $T^{*}=0.09$. (**d**) Competing scaffolds. Similar binding scheme as in (**c**), except that the 2 highest valency proteins now compete for binding to the 3-valency good topology protein (see table inset). In this case, the system condenses into a single droplet, despite the absence of attractive interactions between 4-valency promiscuous proteins and 4-valency selective ones. A representative snapshot of a DC simulation at $T^{*}=0.09$ is shown. The same color code as in a–c is used, except for the 3-valency good topology proteins, which are now depicted in green.

By modulating the number of binding sites, their positions on the protein surface (or the topology), and the strength of binding among sites, our model allows us to vary both the valency of each protein within a multicomponent mixture and the relative binding affinity among protein pairs. The number of different protein components in a mixture can also be arbitrarily varied; hence, the number of potential multicomponent mixtures that we could model is extremely large. Because we are interested in investigating the impact of the variance in valency and binding affinity, we sought to model systems with the largest number of components that were still computationally tractable. Thus, following our previous work [41]—which showed that our model can be efficiently used to investigate multicomponent mixtures containing as much as six different components—we study six-component mixtures. Next, to guarantee that each component is sufficiently different from one another, we combine proteins that exhibit different effective valencies, i.e., decreasing gradually from a value of four (a valency that, within the model energy scale, strongly sustains LLPS when the protein is in pure form) to a value of two (a valency that instead inhibits LLPS when the protein is in pure form) [see Figure 1]. Due to the approximate nature of our model, rather than assessing fine variations in the binding affinities among proteins, we focus on contrasting the limits of high versus low binding affinities among protein pairs. In practice, to model the higher probability of binding among high-affinity pairs, we assign a value of ϵ (the unit of energy in our model; proportional to $k_{B}T$) to the strength of all high-affinity protein–protein interactions, and a value of zero to all low-affinity protein–protein interactions. Note that this is equivalent to setting the binding strength among low-affinity binding proteins to a finite value, as long as it is significantly lower to that of the high-affinity binding pairs. Comparing proteins that bind with low versus high affinity, rather than contrasting specific binding affinity values, allows us to reduce the dimensionality of the parameter space significantly and capture general trends. Despite this huge reduction, for a six-component mixture, we are still left with $2^{21}$ possible scenarios: i.e, all the combinations arising for 21 unique protein pairs that can be defined as exhibiting either a low or high binding affinity. From the more than 2 million possible mixtures we could investigate, we have chosen to analyze four cases that mimic widely different biological scenarios. The four different mixtures include the same components but are distinguished by the relative binding affinities among the components; these mixtures are schematized in Figure 2 and the rationale behind their choice is explained in detail below.

As a control and to isolate the effects of valency from other factors, we first define a mixture where we allow all binding sites, regardless of their parent protein, to interact with one another with high binding affinity. Because the differences in the probabilities that any two proteins in this mixture would bind to one another is exclusively determined by their valencies (i.e., the higher the average valency of two proteins, the higher their binding affinity), we term this control mixture ‘valency-driven binding’ [Figure 2a]. The three additional mixtures explore variations in the binding affinities among the various multivalent proteins; large binding affinity variations are expected to occur within in vivo condensates [90,91]. Specifically, our second mixture was designed to amplify the impact of valency on the binding affinity of proteins. Accordingly, we defined the binding affinity among equal or similar-valency proteins (i.e., Δ valency ≤1) as high, and that among proteins with dissimilar valencies (i.e., Δ valency >1) as low. We refer to this mixture as ‘like-valency binding’ [Figure 2b] to highlight the positive correlation between valency and binding affinity that drives proteins to bind predominantly to species with similar valencies to theirs, and favors homotypic interactions too. That is, lower valency proteins become even poorer competitors for high-valency binding sites, which causes them to interact preferentially with other low-valency proteins. Such preferential binding among higher-valency species has been observed in scaffold–client systems [40]. The next two mixtures represent cases where we have two different types of proteins that can act as independent scaffolds of condensates (i.e., they both exhibit LLPS in pure form in the range of conditions investigated), and that are inert to one another; as before, all proteins can also establish homotypic interactions. Specifically, in the third mixture, named ‘non-competing scaffolds’ [Figure 2c], the two independent scaffolds do not compete strongly to recruit clients, as each scaffold binds with high affinity to a different client and with low affinity to the rest. The last mixture, named ‘competing scaffolds’ [Figure 2d], considers the scenario where the two independent scaffolds compete strongly to recruit a common intermediate-valency client.

**Figure 3:**
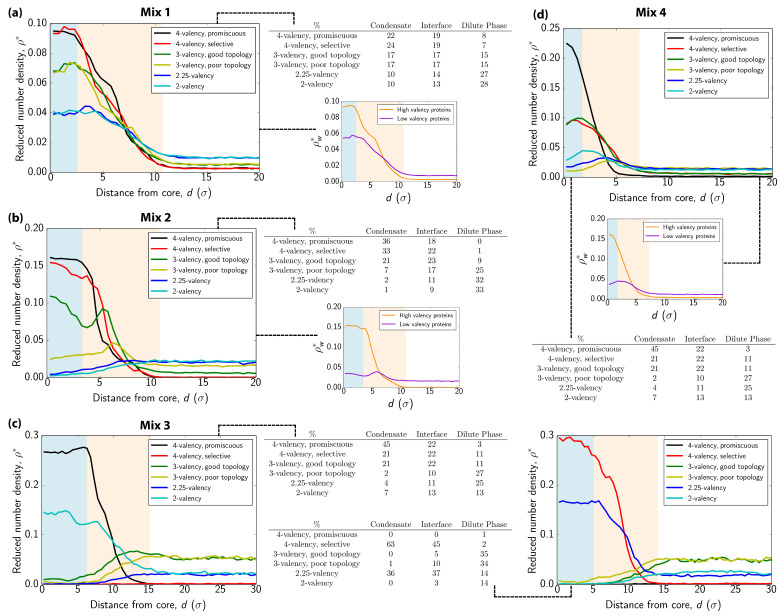
Structural insights of multilayer and multidroplet organization. (**a**–**d**) Density profiles of each protein type for the systems depicted in Figure 2a–d, respectively. In each profile, we plot the reduced density ($\rho^{*}$) of a given protein as a function of distance (in units of molecular diameter, σ) from the droplet center of mass along the perpendicular direction to the interface (long axis of the simulation box). The protein concentration of each system in the condensate (blue shaded region), interfacial boundaries (beige shaded region) and dilute phase (white region) are reported for each mixture. Density profiles of the different mixtures in terms of high-valency proteins (4-valency) vs low-valency ones (3- and 2-valency) are also given for mixtures (**a**,**b**,**d**). Since the number of low-valency species is double than that of high-valency ones by construction, we plot weighted density $\rho_{w}^{*}$ (total density of scaffolds/clients divided by the number of protein types belonging to each family) against distance from the center of mass of the droplet. Note that for mixture (**c**) we display two density profiles corresponding to each of the droplets observed in Figure 2c: (right) the 4-valency promiscuous rich one and (left) the 4-valency selective rich droplet. The temperatures at which these analyses were performed are those indicated in Figure 2.

We observe that our ‘valency-driven binding’ condensates exhibit a modest heterogeneous distribution of their six different protein components [Figure 3a]. That is, when we first measure the relative concentration of each type of protein as we move from the center of the condensate to the interface [Figure 3a], we find that all proteins are present across the whole condensate. However, when we compare the profiles for the highest versus lowest valency proteins, we find that two highest-valency proteins (i.e., the 4-valency promiscuous and selective) are more concentrated at the condensate center [blue shaded region in Figure 3a], and the two lowest valency species (i.e., 2.25-valency and 2-valency) show a slight increase in their concentration at the interface [beige shaded region in Figure 3a]. In addition, as we observed before [41], in these ‘valency-driven binding’ condensates, the relative concentration of a protein within the condensate is positively correlated with its valency [Figure 3a]. Such preferential concentration of higher valency species within condensates is expected to maximize the overall number of LLPS-stabilizing molecular connections per unit of volume and increase the condensate stability [41]. In the following section, we discuss an additional key thermodynamic driving force besides enthalpy leading to the formation of these multilayered condensates.

Interestingly, in our ‘like-valency binding’ condensates, i.e., where proteins bind preferentially to those of similar valencies, a more notable heterogeneous distribution of scaffolds and clients within the condensate emerges [Figure 3b]. Specifically, the ‘like-valency binding’ mixture forms a condensate with a core that is almost exclusively enriched in scaffolds [i.e., the 4-valency promiscuous and selective proteins in black and red, respectively in Figure 3b] and that is mostly surrounded by clients [i.e., lower valency proteins; purple curve in Figure 3b]. Here, the intermediate valency proteins (3-valency good and poor topology) show a clear maximum in concentration near the interfaces, as this facilitates their interactions with high-valency proteins at the core and low-valency proteins at the outer interface [Figure 3b]. Positioning high-valency proteins at the condensate core favors preferential saturation of their binding sites, enhancing the molecular connections per unit of volume of the condensate [41].

Instead of forming a single six-component condensate, the imbalance of interactions between the proteins in our ‘non-competing scaffolds’ mixture leads to the formation of two distinct condensates with completely different compositions [see Figure 2c and Figure 3c]. Notably, each of these droplets has a multilayer organization with a core enriched in one of the two high-valency species—i.e., either 4-valency-promiscuous or 4-valency-selective—and an outer layer consisting of different low-valency species [see Figure 2c and Figure 3c]. Because the intermediate-valency proteins (i.e., the 3-valency good and 3-valency poor topology species) do not interact directly with either of the two high-valency proteins, they remain essentially excluded from both condensates, except at the interfaces where they exhibit a moderate maximum concentration. Despite the expected high energetic cost associated with the formation of two different interfaces, the formation of two separate multilayered condensates becomes thermodynamically stable due to the significant enthalpic gain obtained by burying each type of high valency proteins deep into the core of a separate condensate and, as such, fully saturating their binding sites. We have confirmed that these two different equilibrium condensates are stable at various temperatures, diffuse well, and are immiscible even when in contact in very long simulations ($t^{*}=10^{5}$). This scenario highlights that modulation of the binding affinities among different components, for instance stemming from post-translational modifications or introduction of additional components, can have a strong impact on the spatial organization of proteins within biomolecular condensates, and even provide a mechanism to trigger the fission of condensates into different coexisting drops with diverse compositions.

Surprisingly, our simulations reveal that activating the interaction between the intermediate valency client and the two competing scaffolds, i.e., moving to our ‘competing scaffolds’ mixture, rather than lead to competitive recruitment of the client into the two different condensates, triggers fusion of the two separate condensates into a single drop [Figure 2d]. This new fused multicomponent condensate also exhibits a distinctive heterogeneous distribution of species within [Figure 3d], consisting of an inner core predominantly composed of the highest valency protein in the mixture (i.e, the 4-valency promiscuous protein; black spheres in Figure 2d, and an outer layer enriched in the rest of the species (i.e., the 4-valency selective, 3-valency good topology, and the lower valency species in red, dark green, and grey spheres, respectively in Figure 2d. In the fused condensate, the two highest valency proteins, which are inert to one another, are effectively bridged (but not entirely mixed) by the intermediate-valency protein that binds strongly to both. Indeed, as in the previous multilayered architectures we observe, the low-valency proteins are most concentrated in the outer layers of the condensate, exhibiting a maximum in concentration at the droplet interface (Figure 3d).

**Figure 4:**
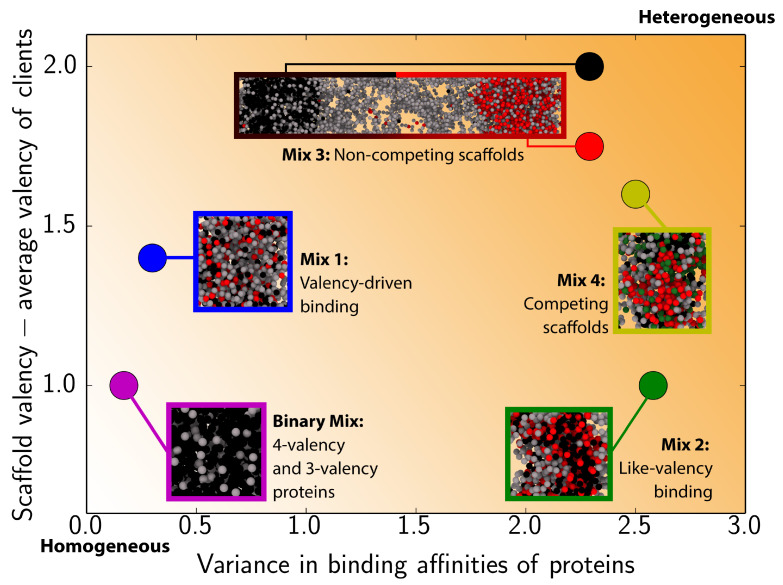
Relationship between valency and binding affinity for the different studied mixtures. On the y-axis, we plot the difference between the scaffold valency and the average valency of clients. Here, scaffolds are: 4-valency promiscuous proteins for the Binary Mix [41] and for the black condensate in Mix 3; both 4-valency proteins (i.e., promiscuous and selective) in Mix 1, 2, and 4; and 4-valency selective proteins for the red condensate in Mix 3. Note, we consider separate scaffolds for Mix 3; since, in that system, the two types of 4-valency proteins form distinct condensates. To calculate the average valency of clients, we only consider clients that can bind to the respective scaffolds with a high binding affinity (i.e., non-zero in this case; Figure 2). The x-axis represents the variance in pairwise binding affinities for all the proteins in the system; where the binding affinity for a given pairwise interaction is taken as the average valency of the two proteins in question (see table insets in Figure 2 for details on pairwise binding interactions). An increase in either the scaffold–client valency difference or the variance in protein binding affinities lead to progressively more heterogeneous condensates, as indicated by the orange shaded background.

As discussed above, the number of potential systems that can be explored with our approach is very large. The limited set that we investigate here, explore scenarios that we believe might be biologically relevant. For instance, the ‘valency-driven binding’ and ‘like-valency binding’ scenarios, look at how variations in the valency, and the subsequent preferential binding among like-valency species, can give rise to different multilayered architectures. These scenarios are likely relevant in systems like stress granules [39,45], the nucleoli [15], and scaffolds-and-clients in vitro systems [40]. Our ‘non-competing scaffolds’ and ‘competing scaffolds’ systems, assess cases where multicomponent systems might form immiscible multilayered droplets that can later be driven to undergo fusion by the addition of a common client. To summarize some of the key differences among the four mixtures we investigate and our findings, for each mixture we define two order parameters that measure the differences in the valencies among scaffolds and strongly-bound clients, and the variance in binding affinities among all protein pairs in the mixtures, respectively. Figure 4 locates our systems within a fraction of the enormous multiparametric phase-space they occupy, and shows how multilayered structures are favoured when the difference in the valencies of scaffolds and clients grows and/or the variance in the binding affinities among components increases.

### 3.2. Interfacial Free Energy as a Driving Force for Multilayered Condensate Organization

A common feature we observe in all the multilayered condensates we study is that low-valency proteins are preferentially positioned towards the interface. Therefore, we investigated if by positioning low-valency proteins at the interface, multilayered condensates incur in a less significant interfacial energetic penalty than what they would exhibit if the high-valency species where placed at the interface instead. To test this hypothesis, following the procedure described in [53], we evaluate the interfacial free energy (or surface tension) of both a single-component condensate formed by the highest valency protein in our set (i.e., 4-valency/promiscuous), and our different six-component condensates, each measured below their respective critical temperatures. We find that by committing low-valency clients (2.25- and 2-valency proteins) to the condensate surface, the ‘valency-driven binding’ multicomponent condensate achieves a huge reduction in its surface tension, with respect to the cost of instead positioning high-valency proteins at the interface (i.e., $\gamma =0.05\pm 0.04k_{B}T/\sigma^{2}$ and $1.44\pm 0.2k_{B}T/\sigma^{2}$ for the six-component condensate versus the single-component 4-valency promiscuous condensate, respectively, both measured at at $T^{*}$ = 0.09). Similarly, the ‘like-valency binding’ condensates with an interface enriched in 3-valency proteins exhibit a much lower surface tension ($\gamma =0.33\pm 0.20k_{B}T/\sigma^{2}$ at $T^{*}$ = 0.09) than the 4-valency promiscuous condensate at the same temperature. Such trend is also evident in the ‘Non-competing scaffolds’ mixture that forms two separate multilayered condensates, each with a lower surface tension ($\gamma =0.2\pm 0.15k_{B}T/\sigma^{2}$ for the droplet rich in 4-valency-promiscuous proteins and $\gamma =0.21\pm 0.1k_{B}T/\sigma^{2}$ for the droplet rich in 4-valency-selective proteins, measured at $T^{*}$ = 0.083) than that of the 4-valency-promiscuous drop at the same temperature (i.e., $1.94\pm 0.2k_{B}T/\sigma^{2}$ at $T^{*}$ = 0.083). Lastly, the ‘Competing scaffolds’ system with various lower valency species at the surface also shows a much lower surface tension ($\gamma =0.09\pm 0.04k_{B}T/\sigma^{2}$ at $T^{*}$ = 0.09) than that of the pure 4-valency promiscuous system ($\gamma =1.44\pm 0.2k_{B}T/\sigma^{2}$ at $T^{*}$ = 0.09). The results of the surface tension for the different mixtures and temperatures are summarized in Table 1. These results—which collectively suggest that multilayered organizations that position the lowest valency proteins towards the interface indeed reduce the interfacial free energy of the condensate—are consistent with previous experimental work suggesting that the surface tension is one of the key factors dictating the multilayered structure of condensates [15,47].

### 3.3. Exchange of Species in and Out of Condensates

**Figure 5:**
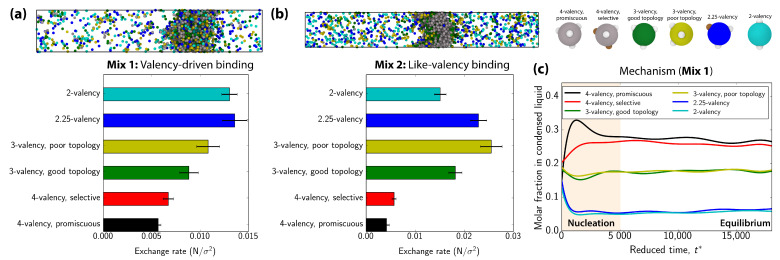
(**a**,**b**) Exchange rate between the condensate and the dilute phase for proteins in systems (**a**) and (**b**) shown in Figure 2 and Figure 3. The exchange rate is defined as the averaged difference in the number of proteins per unit of area (N/$\sigma^{2}$; in our calculations, the interfacial area of the slab is two times the cross-section of our DC simulation box) for each component between subsequent independent configurations. One configuration is considered independent from the previous one when the proteins inside the condensate have diffused at least one molecular diameter. For both mixtures, a snapshot of the direct coexistence simulation from which exchange rate calculations were performed is shown. 4-valency proteins are colored in grey. The coloring code for all other proteins is the same as that used in the exchange rate diagrams. (**c**) Time-evolution of the molar fraction of each individual specie in the condensed liquid phase as a function of time (in reduced units) for the valency-driven system (Mix 1). $t^{*}$ = 0 corresponds to the homogeneous fluid state of the 6-component mixture. The temperature of all the **NVT** simulations shown in this Figure is $T^{*}$ = 0.09.

To understand the potential implications of the inhomogeneous distribution of proteins within multicomponent condensates, we monitor the exchange rate of the proteins with different valencies in and out of the condensates, by defining an exchange rate order parameter. This order parameter describes the average difference in the number of proteins of each type, per unit of droplet area, that are exchanged between the condensate and the dilute phase among subsequent independent configurations. A detailed description of this order parameter is given in Appendix B. Here, a configuration is considered independent from the previous one when the proteins inside the condensate have diffused at least one molecular diameter. Hence, higher values of our exchange rate order parameter imply that proteins leave and reenter the condensate more frequently. Figure 5a,b show a significant difference between the exchange rates of high and low valency proteins for the ‘valency-driven’ and ‘like-valency’ binding systems, respectively. Therefore, an important functional implication stemming from the multilayered architecture of condensates is their ability to selectively control the rate of exchange among different species. Proteins located towards the surface (e.g., lower valency proteins), which negligibly affect condensate stability, can readily exchange in and out of condensates, whereas those essential to decrease the enthalpy of the condensate and maintain its stability (higher valency species) remain buried deep in the condensate core and exhibit sensibly lower exchange rates. We note that, in our systems, the lowest valency proteins among the set (i.e., 2-valency proteins) do not have the highest exchange rate because those proteins are significantly depleted from the condensate throughout the simulations. This result reveals how the exchange rate is correlated with the condensate partitioning coefficient, which depends on the local environment, and which in heterogeneous multiphase condensates is not easily defined. The inhomogeneous exchange of species in and out of condensates is consistent with experiments revealing a much higher diffusion of RFP-SIM (low-valency client) than that of its high-valency scaffold polySUMO [40]. Furthermore, high-valency proteins (PML I–VI proteins) of promyelocytic leukemia nuclear bodies have higher residence times in the condensate than the lower-valency species such as DAXX or BML [94]. Our results also suggest how protein valency might explain the observation that stress granules from stable cores that are surrounded by highly dynamic shells, that exhibit high surface exchange rates with the cytosol [45].

Our simulations further reveal that the differential exchange of low and high valency proteins in and out of condensates is also consistent with the different role that such proteins play in the mechanism of formation of multilayered condensates. To assess such mechanism, we focus on the ‘valency-driven’ mixture and monitor the molar fraction of each component inside the condensate as it nucleates and grows from the solution [Figure 5c]. Consistent with the scaffold–client model of Banani et al. [40], our six-component ‘valency-driven binding’ condensate forms via initial scaffold nucleation, which is followed by subsequent recruitment of clients [40,41]. That is, the first nuclei are mostly composed of the highest valency proteins [see peak at $t^{*}$∼1500 in the 4-valency promiscuous curve (black) of Figure 5c], and as these nuclei grow and fuse, lower valency components are slowly incorporated until an equilibrium composition is reached ($t^{*}$∼5000). Though we have only evaluated this mechanism for the ‘valency-driven’ mixture, we expect this behavior to be even sharper for the other systems where multilayered condensate organization is more pronounced.

## 4. Conclusions

Our simulations reveal how very subtle variations in the valencies and binding affinities among different interacting multivalent proteins can modulate the internal structure and composition of multicomponent condensates. We find that within multicomponent condensates, high-valency proteins are placed preferentially at the core of the droplets, while low-valency species concentrate towards the interface. Positioning the highest valency proteins at the core ensures saturation of their binding sites, and hence, maximizes the total number of bonds per unit of volume of condensate (enthalpy maximization). Simultaneously, positioning the low-valency species towards the surface decreases the energetic penalty for interface formation (interfacial free energy minimization). The key role of the surface tension in driving multilayered condensate organization has been reported for in vitro complex coacervates [47,50]. Moreover, our findings provide a thermodynamic explanation for the scaffold-client model [40], which proposes that nucleation of biomolecular condensates with many components is driven by interactions between high-valency proteins. Our simulations show that, during the first stages of nucleation, the high-valency proteins bind to each other first, yielding small protein clusters. As the high-valency nuclei begin to grow, they recruit lower-valency clients, keeping them around the core, and thus minimizing the interfacial free energy until they reach the equilibrium composition. This further suggests how the concept of valency (and its thermodynamic implications on the condensate) might account on its own for the multilayered organization of systems such as stress granules [39,45], the nucleoli [15], nuclear speckles [46], in vitro complex coacervates [33,47,48], and mixtures of RNA-binding proteins and RNA molecules [34,49].

Our work also suggests mechanisms to regulate fission of condensates into multiple drops with varying composition and fusion of various drops into a single multilayered condensate. When two different high-valency proteins in a multicomponent mixture are inert to one another and can concurrently act as scaffolds, they can each nucleate a separate condensate independently and recruit different types of clients, forming two different stable multilayered liquid condensates with distinct compositions. However, the addition of a client that is capable of interacting strongly with both types of scaffolds can induce fusion of the two droplets into a single (highly heterogeneous) condensate. Hence, the addition of clients or chemical modifications on the binding affinities between clients and scaffolds can be crucial for the organization of multicomponent condensates, including those where high-valency proteins are inert to one another but can be bridged by clients. For instance, plant-specific protein Embryo Defective 1579 condensates—implicated in regulating plant gene transcription, mRNA splicing, growth and development—use the DNA Damage Binding Protein 1 (DDB1) and Cullin 4 (CUL4) complex as molecular bridge to recruit CURLY LEAF-containing Polycomb Repressive Complex 2 into the condensates [90]. This ability of protein liquid droplets to easily fuse or split on demand based on the activation/deactivation of interactions among their members, or the introduction of new species, might be a mechanism used by cells to control the spatial segregation of their components [54,55].

Finally, we note that in our model, the absence of an explicit solvent implies that, by construction, low-valency proteins will show lower interfacial free energy than high-valency ones. This is a reasonable approximation since high-valency proteins are usually expected to present higher pure component critical points and higher surface tensions with the solvent than low-valency proteins [95] (or engineered peptides [50]). However, high-valency proteins with specific domains enriched in hydrophilic residues, still could exhibit lower interfacial free energy with the solvent than low-valency hydrophobic species. Nonetheless, even in that concrete scenario, the interplay between the enthalpic gain for binding site saturation by high-valency proteins (or domains) and the surface tension minimization of the total free energy upon condensate formation, might still favor burying the high-valency proteins (or sequence domains) deep down in the condensate core and exposing the low-valency species towards the surface. In this study, we focus on the effect that binding affinities between different multivalent proteins has on LLPS; although we note, that our results are subjected to a key magnitude in LLPS of multicomponent condensates that is, the surface volume ratio of the droplets (in our simulations, the cross-section of the simulation box and the total number of proteins in the system). This important factor which modulates the appearance of multilayered and/or multidroplet condensates when several species are present, will be further investigated in future work. Taken together, our work highlights how subtle changes in binding affinities between proteins in a multicomponent mixture can crucially transform the molecular organization and multilayer behavior of biomolecular condensates.

## Figures and Tables

**Table 1:** Surface tension (γ) of the pure component 4-valency promiscuous scaffold protein and the four multicomponent mixtures studied in this work. Note that we provide two results for the Non-competing scaffolds mixture, one for the 4-valency promiscuous droplet (left) and another for the 4-valency selective condensate (right). The surface tension has been calculated following the methodology detailed in references [92,93].

	Surface Tension, $\gamma (k_{B}T/\sigma^{2})$	
**System**	$T^{*}=0.09$	$T^{*}=0.083$
Pure component 4-valency promiscuous	1.44 ± 0.2	1.94 ± 0.2
Valency-driven binding mixture	0.05 ± 0.04	-
Like-valency binding mixture	0.33 ± 0.20	-
Non-competing scaffolds mixture	-	0.2 ± 0.15 & 0.21 ± 0.1
Competing scaffolds mixture	0.09 ± 0.04	-

**Figure A1:**
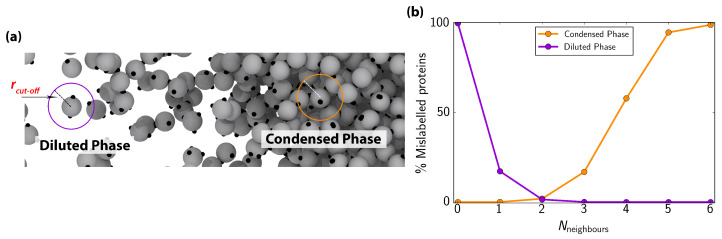
(**a**) Snapshot of the interfacial region between the two coexisting phases of a 6-component mixture. The order parameter distinguishes between proteins (in grey) of both phases by evaluating the number of neighbours that each protein has for a given cut-off distance. For considering a protein belonging to the condensed phase, it needs to have at least 2 neighbours (independent of its valency) within a cut-off distance of $r_{\mathrm{cut}-\mathrm{off}}=1.20\sigma$. (**b**) Percentage of mislabelled proteins as a function of the number of neighbours in the diluted (purple curve) and in the condensed phase (orange curve) for a cut-off distance of 1.20σ. Both curves (phases) were evaluated in bulk NVT simulations at the coexisting density and composition of the ‘valency-driven binding’ system at $T^{*}$ = 0.09.

## Data Availability

The source code for the patchy-particle model can be accessed here: https://github.com/CollepardoLab/md_patchy_model.

## Appendix A. Simulation Details

To perform our calculations, we use molecular dynamics (MD) simulations. We model our coarse-grained multivalent proteins using the MD-Patchy model proposed in Ref. [53], which requires two different set of potentials: a Pseudo Hard-Sphere (PHS) [96] potential to continuously describe the repulsive interaction and excluded volume between Hard-Spheres, and a continuous square-well (CSW) [97] potential for the patch-patch attractive interactions. The $u_{PHS}$ is given by the following expression:

(A1) $$u_{PHS}=\left\{\begin{array}{cc} \lambda_{r}{\left(\frac{\lambda_{r}}{\lambda_{a}}\right)}^{\lambda_{a}}\epsilon_{R}\left[{\left(\frac{\sigma }{r}\right)}^{\lambda_{r}}-{\left(\frac{\sigma }{r}\right)}^{\lambda_{a}}\right]+\epsilon_{R}; & \mathrm{if}\;r<(\frac{\lambda_{r}}{\lambda_{a}})\sigma \\ 0; & \mathrm{if}\;r\geq (\frac{\lambda_{r}}{\lambda_{a}})\sigma \end{array}\right.$$

where $\lambda_{a}=49$ and $\lambda_{r}=50$ are the exponents for the attractive and repulsive terms respectively, $\epsilon_{R}$ accounts for the energy shift of the PHS interaction, σ is the molecular diameter (and our unit of length) and *r* is the center-to-center distance between different PHS particles. For the patch–patch interaction we use the following expression:

(A2) $$u_{CSW}=-\frac{1}{2}\epsilon_{CSW}\left[1-\tanh(\frac{r-r_{w}}{\alpha })\right]$$

where $\epsilon_{CSW}$ is the depth of the potential energy well (and also the unit of energy in our simulations), $r_{w}$ the radius of the attractive well, and α controls the steepness of the well. We choose α=0.005σ and $r_{w}=0.12\sigma$ so that each patch can only interact with another single patch.

The mass of each patch is a 5% of the central particle, which is set to $3.32\times 10^{26}$ kg, despite being this choice irrelevant for equilibrium simulations. This 5% ratio fixes the moment of inertia of the patchy particles. All our results are given in reduced units: reduced temperature is defined as $T^{*}=k_{B}T/\epsilon_{CSW}$, reduced density as $\rho^{*}=\left(N/V\right)\sigma^{3}$, reduced pressure as $p^{*}=p\sigma^{3}/\left(k_{B}T\right)$, and reduced time as $t^{*}=t\sqrt{\sigma^{2}m/\left(k_{B}T\right)}$. In order to keep the PHS interaction as similar as possible to a pure HS interaction, we fix $k_{B}T/\epsilon_{R}$ at a value of 1.5 as suggested in Ref. [96]. We then control the effective strength of the attraction by varying $\epsilon_{CSW}$ such that the reduced temperature $T^{*}=k_{B}T/\epsilon_{CSW}$ is of the order of O(0.1).

Since $u_{PHS}$ and $u_{CSW}$ potentials are both continuous and differentiable, we perform all our simulations using the LAMMPS Molecular Dynamics package [98]. To carry out our two-phase coexisting simulations, we employ the direct coexistence method [40,88,89]. This method consists in simulating coexistence by preparing periodically extended slabs of the two coexisting phases, the condensed and the diluted one. We use an implicit solvent model; accordingly, the diluted-liquid phase (protein-poor phase) and the condensed-liquid phase (protein-rich phase) are effectively a vapour and a liquid, respectively. We prepare the initial configurations in the following way: First, we build a cubic box configuration containing all the components that we want to consider in our mixture. Then, we run an NpT simulation so that our system condenses. For that purpose, conditions of temperature $T^{*}=0.09$ and pressure $p^{*}=0.16$ are enough. We then elongate the box in one direction (say, *x*) by performing an $Np_{x}T$ simulation at $T^{*}=0.09$ and $p^{*}=-0.16$ until the *x*-length of the simulation box is around 6 times longer than the other two. As a final step in the preparation, we run an NVT simulation at $T^{*}=0.15$, temperature at which all proteins distribute homogeneously in the simulation box. Then, this well-mixed system is used as an initial configuration for all the simulations, which are conducted in the NVT ensemble.

For all mixtures we used 1800 proteins, i.e., 300 proteins of each type, as previously described. The resulting sizes of the simulation boxes were: $L_{x}=6L_{y}=6L_{z}=89.5\sigma$ for Mix 1 (‘valency-driven binding’); $L_{x}=6.6L_{y}=6.6L_{z}=88.5\sigma$ for Mix 2 (‘like-valency binding’), $L_{x}=8.2L_{y}=8.2L_{z}=129\sigma$ for Mix 3 (‘Non-competing scaffolds’), and $L_{x}=6L_{y}=6L_{z}=89.5\sigma$ for Mix 4 (‘Competing scaffolds’). Periodic boundary conditions were used in the three directions of space. The timestep chosen for the Verlet integration of equations of motion was $\Delta t^{*}$ = 3.7 × 10^−4^. The cut-off radius was set to 1.17σ for both potentials. We use a Nosé-Hoover thermostat [99,100] for the NVT simulations with a relaxation time of 0.074 in reduced units. For NpT simulations, a Nosé-Hoover barostat with the same relaxation time was employed. To compute the phase diagram and interfacial free energy from our direct coexistence simulations we employ the methodology explained in Ref. [53]

## Appendix B. Local Order Parameter

To identify the number of proteins that belong to the condensed or to the diluted phase as in Figure 5, we make use of a simple local order parameter [101,102,103] based on the number of neighbours that a protein has within a given cut-off distance [see Figure A1a].

To tune the order parameter, we need to carefully define a number of neighbours threshold that, for a given cut-off distance, allows us to fairly identify which proteins belong to each phase. To determine such threshold we use the following procedure: For setting the cut-off distance, we compute the radial distribution function of the 6-component mixture in the condensed phase at the coexisting density and composition at $T^{*}=0.09$. We select a slightly larger distance of the first minimum of the radial distribution function, which in our case is $r_{c}=1.20\sigma$ (the first minimum in the radial distribution function roughly appears at 1.17σ). Secondly, for determining the threshold in the number of neighbours for such cut-off distance, we simulate both phases independently at their equilibrium coexisting conditions and we evaluate the number of mislabelled proteins as a function of different number of neighbours (i.e., 0, 1, 2, …, 6) [see Figure A1b]. The number of neighbours which minimizes the percentage of mislabelled proteins in both phases is then selected as a threshold. Our local order parameter threshold identifies that any protein with two or more neighbours within a cut-off distance of $r_{c}=1.20\sigma$ should be considered as a condensed-liquid-like protein ($N_{Neighbours}\geq 2$). Although for our purposes the optimization of the order parameter is not crucial, since these values was used as an input for obtaining any quantitative prediction from theories such as the Classical Nucleation Theory [104,105,106], this criterion allows us to reliably identify the number of proteins composing each phase in coexisting liquid-vapor systems [101,103]. Lastly, to compute the different density profiles shown in Figure 3, we employ this order parameter to find the biggest cluster of proteins belonging to the condensed phase and then find its center of mass employing an algorithm than allows us to determine such center through the periodic boundary conditions in the long axis of the simulation box [107].

## References

[1] Sear R.P. (2005). The cytoplasm of living cells: A functional mixture of thousands of components. J. Phys. Condens. Matter.

[2] Hyman A.A., Weber C.A., Jülicher F. (2014). Liquid-Liquid Phase Separation in Biology. Annu. Rev. Cell Dev. Biol..

[3] Banani S.F., Lee H.O., Hyman A.A., Rosen M.K. (2017). Biomolecular condensates: Organizers of cellular biochemistry. Nat. Rev. Mol. Cell Biol..

[4] Shin Y., Brangwynne C.P. (2017). Liquid phase condensation in cell physiology and disease. Science.

[5] Su X., Ditlev J.A., Hui E., Xing W., Banjade S., Okrut J., King D.S., Taunton J., Rosen M.K., Vale R.D. (2016). Phase separation of signaling molecules promotes T cell receptor signal transduction. Science.

[6] Li P., Banjade S., Cheng H., Kim S., Chen B., Guo L., Llaguno M., Hollingsworth J.V., King D.S. (2012). Phase transitions in the assembly of multivalent signalling proteins. Nature.

[7] Shin Y., Chang Y.C., Lee D.S., Berry J., Sanders D.W., Ronceray P., Wingreen N.S., Haataja M., Brangwynne C.P. (2018). Liquid Nuclear Condensates Mechanically Sense and Restructure the Genome. Cell.

[8] Sabari B.R., Dall’Agnese A., Boija A., Klein I.A., Coffey E.L., Shrinivas K., Abraham B.J., Hannett N.M., Zamudio A.V., Manteiga J.C. (2018). Coactivator condensation at super-enhancers links phase separation and gene control. Science.

[9] Klosin A., Oltsch F., Harmon T., Honigmann A., Jülicher F., Hyman A.A., Zechner C. (2020). Phase separation provides a mechanism to reduce noise in cells. Science.

[10] Sheu-Gruttadauria J., MacRae I.J. (2018). Phase Transitions in the Assembly and Function of Human miRISC. Cell.

[11] Franzmann T.M., Alberti S. (2019). Prion-like low-complexity sequences: Key regulators of protein solubility and phase behavior. J. Biol. Chem..

[12] Kroschwald S., Munder M.C., Maharana S., Franzmann T.M., Richter D., Ruer M., Hyman A.A., Alberti S. (2018). Different Material States of Pub1 Condensates Define Distinct Modes of Stress Adaptation and Recovery. Cell Rep..

[13] Bouchard J.J., Otero J.H., Scott D.C., Szulc E., Martin E.W., Sabri N., Granata D., Marzahn M.R., Lindorff-Larsen K., Salvatella X. (2018). Cancer Mutations of the Tumor Suppressor SPOP Disrupt the Formation of Active, Phase-Separated Compartments. Mol. Cell.

[14] Alberti S., Carra S. (2018). Quality Control of Membraneless Organelles. J. Mol. Biol..

[15] Feric M., Vaidya N., Harmon T.S., Mitrea D.M., Zhu L., Richardson T.M., Kriwacki R.W., Pappu R.V., Brangwynne C.P. (2016). Coexisting liquid phases underlie nucleolar subcompartments. Cell.

[16] Lee K.H., Zhang P., Kim H.J., Mitrea D.M., Sarkar M., Freibaum B.D., Cika J., Coughlin M., Messing J., Molliex A. (2016). C9orf72 Dipeptide Repeats Impair the Assembly, Dynamics, and Function of Membrane-Less Organelles. Cell.

[17] Mitrea D.M., Cika J.A., Guy C.S., Ban D., Banerjee P.R., Stanley C.B., Nourse A., Deniz A.A., Kriwacki R.W. (2016). Nucleophosmin integrates within the nucleolus via multi-modal interactions with proteins displaying R-rich linear motifs and rRNA. eLife.

[18] Woodruff J.B., Gomes B.F., Widlund P.O., Mahamid J., Honigmann A., Hyman A.A. (2017). The Centrosome Is a Selective Condensate that Nucleates Microtubules by Concentrating Tubulin. Cell.

[19] Alberti S., Gladfelter A., Mittag T. (2019). Considerations and challenges in studying liquid–liquid phase separation and biomolecular condensates. Cell.

[20] Brundin P., Melki R., Kopito R., Brundin P., Melki R., Kopito R. (2010). Prion-like transmission of protein aggregates in neurodegenerative diseases. Nat. Rev. Mol. Cell Biol..

[21] Ambadipudi S., Biernat J., Riedel D., Mandelkow E., Zweckstetter M. (2017). Liquid–liquid phase separation of the microtubule-binding repeats of the Alzheimer-related protein Tau. Nat. Commun..

[22] Shulman J.M., De Jager P.L., Feany M.B. (2011). Parkinson’s Disease: Genetics and Pathogenesis. Annu. Rev. Pathol. Mech. Dis..

[23] Ray S., Singh N., Kumar R., Patel K., Pandey S., Datta D., Mahato J., Panigrahi R., Navalkar A., Mehra S. (2020). *α*-Synuclein aggregation nucleates through liquid–liquid phase separation. Nat. Chem..

[24] Robberecht W., Philips T. (2013). The changing scene of amyotrophic lateral sclerosis. Nat. Rev. Neurosci..

[25] Molliex A., Temirov J., Lee J., Coughlin M., Kanagaraj A., Kim H., Mittag T., Taylor J. (2015). Phase Separation by Low Complexity Domains Promotes Stress Granule Assembly and Drives Pathological Fibrillization. Cell.

[26] Xiang S., Kato M., Wu L., Lin Y., Ding M., Zhang Y., Yu Y., McKnight S. (2015). The LC Domain of hnRNPA2 Adopts Similar Conformations in Hydrogel Polymers, Liquid-like Droplets, and Nuclei. Cell.

[27] Elbaum-Garfinkle S., Kim Y., Szczepaniak K., Chen C.C.H., Eckmann C.R., Myong S., Brangwynne C.P. (2015). The disordered P granule protein LAF-1 drives phase separation into droplets with tunable viscosity and dynamics. Proc. Natl. Acad. Sci. USA.

[28] Mitrea D.M., Cika J.A., Stanley C.B., Nourse A., Onuchic P.L., Banerjee P.R., Phillips A.H., Park C.G., Deniz A.A., Kriwacki R.W. (2018). Self-interaction of NPM1 modulates multiple mechanisms of liquid–liquid phase separation. Nat. Commun..

[29] Asherie N., Pande J., Lomakin A., Ogun O., Hanson S.R., Smith J.B., Benedek G.B. (1998). Oligomerization and phase separation in globular protein solutions. Biophys. Chem..

[30] Sun X.S., Wang D., Zhang L., Mo X., Zhu L. (2008). Morphology and phase separation of hydrophobic clusters of soy globular protein polymers. Macromol. Biosci..

[31] Joseph J.A., Espinosa J.R., Sanchez-Burgos I., Garaizar A., Frenkel D., Collepardo-Guevara R. (2021). Thermodynamics and kinetics of phase separation of protein–RNA mixtures by a minimal model. Biophys. J..

[32] Burke K.A., Janke A.M., Rhine C.L., Fawzi N.L. (2015). Residue-by-Residue View of In Vitro FUS Granules that Bind the C-Terminal Domain of RNA Polymerase II. Mol. Cell.

[33] Boeynaems S., Holehouse A.S., Weinhardt V., Kovacs D., Van Lindt J., Larabell C., Van Den Bosch L., Das R., Tompa P.S., Pappu R.V. (2019). Spontaneous driving forces give rise to protein-RNA condensates with coexisting phases and complex material properties. Proc. Natl. Acad. Sci. USA.

[34] Sanders D.W., Kedersha N., Lee D.S., Strom A.R., Drake V., Riback J.A., Bracha D., Eeftens J.M., Iwanicki A., Wang A. (2020). Competing protein-RNA interaction networks control multiphase intracellular organization. Cell.

[35] Agrawal S., Kuo P.H., Chu L.Y., Golzarroshan B., Jain M., Yuan H.S. (2019). RNA recognition motifs of disease-linked RNA-binding proteins contribute to amyloid formation. Sci. Rep..

[36] Roden C., Gladfelter A.S. (2020). RNA contributions to the form and function of biomolecular condensates. Nat. Rev. Mol. Cell Biol..

[37] Loughlin F.E., Wilce J.A. (2019). TDP-43 and FUS—Structural insights into RNA recognition and self-association. Curr. Opin. Struct. Biol..

[38] Polymenidou M. (2018). The RNA face of phase separation. Science.

[39] Guillén-Boixet J., Kopach A., Holehouse A.S., Wittmann S., Jahnel M., Schlüssler R., Kim K., Trussina I.R., Wang J., Mateju D. (2020). RNA-induced conformational switching and clustering of G3BP drive stress granule assembly by condensation. Cell.

[40] Banani S.F., Rice A.M., Peeples W.B., Lin Y., Jain S., Parker R., Rosen M.K. (2016). Compositional Control of Phase-Separated Cellular Bodies. Cell.

[41] Espinosa J.R., Joseph J.A., Sanchez-Burgos I., Garaizar A., Frenkel D., Collepardo-Guevara R. (2020). Liquid network connectivity regulates the stability and composition of biomolecular condensates with many components. Proc. Natl. Acad. Sci. USA.

[42] Alberti S. (2017). Phase separation in biology. Curr. Biol..

[43] Wang J., Choi J.M., Holehouse A.S., Lee H.O., Zhang X., Jahnel M., Maharana S., Lemaitre R., Pozniakovsky A., Drechsel D. (2018). A molecular grammar governing the driving forces for phase separation of prion-like RNA binding proteins. Cell.

[44] Nott T., Petsalaki E., Farber P., Jervis D., Fussner E., Plochowietz A., Craggs T.D., Bazett-Jones D., Pawson T., Forman-Kay J. (2015). Phase Transition of a Disordered Nuage Protein Generates Environmentally Responsive Membraneless Organelles. Mol. Cell.

[45] Jain S., Wheeler J.R., Walters R.W., Agrawal A., Barsic A., Parker R. (2016). ATPase-modulated stress granules contain a diverse proteome and substructure. Cell.

[46] Fei J., Jadaliha M., Harmon T.S., Li I.T.S., Hua B., Hao Q., Holehouse A.S., Reyer M., Sun Q., Freier S.M. (2017). Quantitative analysis of multilayer organization of proteins and RNA in nuclear speckles at super resolution. J. Cell Sci..

[47] Lu T., Spruijt E. (2020). Multiphase complex coacervate droplets. J. Am. Chem. Soc..

[48] Mountain G.A., Keating C.D. (2019). Formation of Multiphase Complex Coacervates and Partitioning of Biomolecules within them. Biomacromolecules.

[49] Kaur T., Raju M., Alshareedah I., Davis R.B., Potoyan D.A., Banerjee P.R. (2020). Sequence-encoded and composition-dependent protein-RNA interactions control multiphasic condensate topologies. bioRxiv.

[50] Fisher R.S., Elbaum-Garfinkle S. (2020). Tunable multiphase dynamics of arginine and lysine liquid condensates. Nat. Commun..

[51] Jacobs W.M., Frenkel D. (2017). Phase transitions in biological systems with many components. Biophys. J..

[52] Dar F., Pappu R.V. (2020). Multidimensional Phase Diagrams for Multicomponent Systems Comprising Multivalent Proteins. Biophys. J..

[53] Espinosa J.R., Garaizar A., Vega C., Frenkel D., Collepardo-Guevara R. (2019). Breakdown of the law of rectilinear diameter and related surprises in the liquid-vapor coexistence in systems of patchy particles. J. Chem. Phys..

[54] Wheeler R.J., Hyman A.A. (2018). Controlling compartmentalization by non-membrane-bound organelles. Philos. Trans. R. Soc. Biol. Sci..

[55] Strom A.R., Brangwynne C.P. (2019). The liquid nucleome—Phase transitions in the nucleus at a glance. J. Cell Sci..

[56] Paloni M., Bailly R., Ciandrini L., Barducci A. (2020). Unraveling Molecular Interactions in Liquid–Liquid Phase Separation of Disordered Proteins by Atomistic Simulations. J. Phys. Chem. B.

[57] Zheng W., Dignon G.L., Jovic N., Xu X., Regy R.M., Fawzi N.L., Kim Y.C., Best R.B., Mittal J. (2020). Molecular Details of Protein Condensates Probed by Microsecond Long Atomistic Simulations. J. Phys. Chem. B.

[58] Welsh T.J., Krainer G., Espinosa J.R., Joseph J.A., Sridhar A., Jahnel M., Arter W.E., Saar K.L., Alberti S., Collepardo- Guevara R. (2020). Single particle zeta-potential measurements reveal the role of electrostatics in protein condensate stability. bioRxiv.

[59] Nguemaha V., Zhou H.X. (2018). Liquid-Liquid Phase Separation of Patchy Particles Illuminates Diverse Effects of Regulatory Components on Protein Droplet Formation. Sci. Rep..

[60] Dignon G.L., Zheng W., Mittal J. (2019). Simulation methods for liquid–liquid phase separation of disordered proteins. Curr. Opin. Chem. Eng..

[61] Pak A.J., Voth G.A. (2018). Advances in coarse-grained modeling of macromolecular complexes. Curr. Opin. Struct. Biol..

[62] Ruff K.M., Pappu R.V., Holehouse A.S. (2019). Conformational preferences and phase behavior of intrinsically disordered low complexity sequences: Insights from multiscale simulations. Curr. Opin. Struct. Biol..

[63] Brangwynne C.P., Tompa P., Pappu R.V. (2015). Polymer physics of intracellular phase transitions. Nat. Phys..

[64] Liu H., Kumar S.K., Sciortino F. (2007). Vapor-liquid coexistence of patchy models: Relevance to protein phase behavior. J. Chem. Phys..

[65] Chou H.Y., Aksimentiev A. (2020). Single-Protein Collapse Determines Phase Equilibria of a Biological Condensate. J. Phys. Chem. Lett..

[66] Ruff K.M., Harmon T.S., Pappu R.V. (2015). CAMELOT: A machine learning approach for coarse-grained simulations of aggregation of block-copolymeric protein sequences. J. Chem. Phys..

[67] Harmon T.S., Holehouse A.S., Rosen M.K., Pappu R.V. (2017). Intrinsically disordered linkers determine the interplay between phase separation and gelation in multivalent proteins. eLife.

[68] Choi J.M., Dar F., Pappu R.V. (2019). LASSI: A lattice model for simulating phase transitions of multivalent proteins. PLoS Comput. Biol..

[69] Garaizar A., Sanchez-Burgos I., Collepardo-Guevara R., Espinosa J.R. (2020). Expansion of Intrinsically Disordered Proteins Increases the Range of Stability of Liquid–Liquid Phase Separation. Molecules.

[70] Krainer G., Welsh T.J., Joseph J.A., Espinosa J.R., Wittmann S., de Csilléry E., Sridhar A., Toprakcioglu Z., Gudiškytė G., Czekalska M.A. (2021). Reentrant liquid condensate phase of proteins is stabilized by hydrophobic and non-ionic interactions. Nat. Comms..

[71] Lichtinger S.M., Garaizar A., Collepardo-Guevara R., Reinhardt A. (2020). Targeted modulation of protein liquid–liquid phase separation by evolution of amino-acid sequence. bioRxiv.

[72] Schreck J.S., Romano F., Zimmer M.H., Louis A.A., Doye J.P. (2016). Characterizing DNA star-tile-based nanostructures using a coarse-grained model. ACS Nano.

[73] Doye J.P., Ouldridge T.E., Louis A.A., Romano F., Šulc P., Matek C., Snodin B.E., Rovigatti L., Schreck J.S., Harrison R.M. (2013). Coarse-graining DNA for simulations of DNA nanotechnology. Phys. Chem. Chem. Phys..

[74] Benayad Z., von Bülow S., Stelzl L.S., Hummer G. (2021). Simulation of FUS protein condensates with an adapted coarse-grained model. J. Chem. Theory Comput..

[75] Bianchi E., Largo J., Tartaglia P., Zaccarelli E., Sciortino F. (2006). Phase diagram of patchy colloids: Towards empty liquids. Phys. Rev. Lett..

[76] Martin E.W., Holehouse A.S., Peran I., Farag M., Incicco J.J., Bremer A., Grace C.R., Soranno A., Pappu R.V., Mittag T. (2020). Valence and patterning of aromatic residues determine the phase behavior of prion-like domains. Science.

[77] Russo J., Tartaglia P., Sciortino F. (2009). Reversible gels of patchy particles: Role of the valence. J. Chem. Phys..

[78] Russo J., Tartaglia P., Sciortino F. (2010). Association of limited valence patchy particles in two dimensions. Soft Matter.

[79] Statt A., Casademunt H., Brangwynne C.P., Panagiotopoulos A.Z. (2020). Model for disordered proteins with strongly sequence-dependent liquid phase behavior. J. Chem. Phys..

[80] Dignon G.L., Zheng W., Kim Y.C., Best R.B., Mittal J. (2018). Sequence determinants of protein phase behavior from a coarse-grained model. PLoS Comput. Biol..

[81] Lin Y.H., Forman-Kay J.D., Chan H.S. (2018). Theories for Sequence-Dependent Phase Behaviors of Biomolecular Condensates. Biochemistry.

[82] Hazra M., Levy Y. (2020). Charge pattern affects the structure and dynamics of polyampholyte condensates. Phys. Chem. Chem. Phys..

[83] Blas F.J., Galindo A., Vega C. (2003). Study of the solid-liquid-vapour phase equilibria of flexible chain molecules using Wertheim’s thermodynamic perturbation theory. Mol. Phys..

[84] Kato M., Han T.W., Xie S., Shi K., Du X., Wu L.C., Mirzaei H., Goldsmith E.J., Longgood J., Pei J. (2012). Cell-free formation of RNA granules: Low complexity sequence domains form dynamic fibers within hydrogels. Cell.

[85] Stukowski A. (2009). Visualization and analysis of atomistic simulation data with OVITO—The Open Visualization Tool. Model. Simul. Mater. Sci. Eng..

[86] Rowlinson J.S., Widom B. (2013). Molecular Theory of Capillarity.

[87] Ponomarenko E.A., Poverennaya E.V., Ilgisonis E.V., Pyatnitskiy M.A., Kopylov A.T., Zgoda V.G., Lisitsa A.V., Archakov A.I. (2016). The Size of the Human Proteome: The Width and Depth. Int. J. Anal. Chem..

[88] García Fernández R., Abascal J.L.F., Vega C. (2006). The melting point of ice Ih for common water models calculated from direct coexistence of the solid-liquid interface. J. Chem. Phys..

[89] Espinosa J.R., Sanz E., Valeriani C., Vega C. (2013). On fluid-solid direct coexistence simulations: The pseudo-hard sphere model. J. Chem. Phys..

[90] Zhang Y., Li Z., Chen N., Huang Y., Huang S. (2020). Phase separation of Arabidopsis EMB1579 controls transcription, mRNA splicing, and development. PLoS Biol..

[91] Pazhouhandeh M., Molinier J., Berr A., Genschik P. (2011). MSI4/FVE interacts with CUL4–DDB1 and a PRC2-like complex to control epigenetic regulation of flowering time in Arabidopsis. Proc. Natl. Acad. Sci. USA.

[92] Kirkwood J.G., Buff F.P. (1949). The statistical mechanical theory of surface tension. J. Chem. Phys..

[93] Vega C., De Miguel E. (2007). Surface tension of the most popular models of water by using the test-area simulation method. J. Chem. Phys..

[94] Weidtkamp-Peters S., Lenser T., Negorev D., Gerstner N., Hofmann T.G., Schwanitz G., Hoischen C., Maul G., Dittrich P., Hemmerich P. (2008). Dynamics of component exchange at PML nuclear bodies. J. Cell Sci..

[95] Ijavi M., Style R.W., Emmanouilidis L., Kumar A., Meier S.M., Torzynski A.L., Allain F.H., Barral Y., Steinmetz M.O., Dufresne E.R. (2020). Surface tensiometry of phase separated protein and polymer droplets by the sessile drop method. Soft Matter.

[96] Jover J., Haslam A.J., Galindo A., Jackson G., Müller E.A. (2012). Pseudo hard-sphere potential for use in continuous molecular-dynamics simulation of spherical and chain molecules. J. Chem. Phys..

[97] Espinosa J., Vega C., Sanz E. (2014). The mold integration method for the calculation of the crystal-fluid interfacial free energy from simulations. J. Chem. Phys..

[98] Plimpton S. (1995). Fast Parallel Algorithms for Short-Range Molecular Dynamics. J. Comput. Phys..

[99] Nosé S. (1984). A unified formulation of the constant temperature molecular dynamics methods. J. Chem. Phys..

[100] Hoover W.G. (1985). Canonical dynamics: Equilibrium phase-space distributions. Phys. Rev. A.

[101] ten Wolde P.R., Frenkel D. (1998). Computer simulation study of gas–iquid nucleation in a Lennard-Jones system. J. Chem. Phys..

[102] ten Wolde P.R., Frenkel D. (1998). Numerical study of gas–liquid nucleation in partially miscible binary mixtures. J. Chem. Phys..

[103] Sanchez-Burgos I., de Hijes P.M., Rosales-Pelaez P., Vega C., Sanz E. (2020). Condensation and boiling in a Lennard-Jones fluid. Phys. Rev. E.

[104] Kashchiev D. (2000). Nucleation.

[105] Kelton K.F. (1991). Crystal nucleation in liquids and glasses. Solid State Physics.

[106] Espinosa J.R., Vega C., Valeriani C., Sanz E. (2016). Seeding approach to crystal nucleation. J. Chem. Phys..

[107] Bai L., Breen D. (2008). Calculating center of mass in an unbounded 2D environment. J. Graph. Tools.

